# Intracranial otogenic complications in adults: new factors that influenced its onset, frequency and nature

**DOI:** 10.1186/s40463-021-00554-6

**Published:** 2022-03-04

**Authors:** Małgorzata Wierzbicka, Michalina Staśkiewicz, Oskar Rosiak, Katarzyna Karmelita-Katulska, Witold Szyfter, Wojciech Gawęcki

**Affiliations:** 1 Department of Otolaryngology, Head and Neck Surgery, Poznan University of Medical Sciences, Przybyszewskiego 49, 60-355 Poznan, Poland; 2 Balance Disorders Unit, Department of Otolaryngology, Medical University of Lodz, The Norbert Barlicki Memorial Teaching Hospital, Kopcińskiego 22, 90-153 Lodz, Poland; 3 Department of General Radiology and Neuroradiology, Poznan University of Medical Sciences, Przybyszewskiego 49, 60-355 Poznan, Poland

**Keywords:** COVID-19, SARS-CoV-2, Latent otitis media, Otogenic complication, Cerebral venous thrombosis, Sigmoid sinus thrombosis

## Abstract

**Background:**

To compare the clinical features of two time cohorts of patients: “pre-COVID-19” and “COVID-19”—admitted as emergency with intracranial otogenic complications, with special regard to sigmoid sinus thrombosis (CVST).

**Methods:**

Retrospective analysis of patients documentation concerning urgent procedures of intracranial otogenic complications at tertiary-referral otolaryngology department. Analysed database—pre-COVID-19 cohort (January–February 2019/2020): 1434 otological outpatient visits, 509 planned otosurgeries and 17 urgent otological procedures; COVID-19 cohort (March–April 2020/2021): 1150, 566 and 20 respectively. Overall intracranial complications: 5 and 9 respectively. Analysed outcome measures: incidence proportion of otogenic intracranial complications in relation to planned and urgent otosurgical procedures; incidence proportion of intracranial complications in relation to the total number of emergency and planned outpatient consultations and the total number of planned surgical procedures.

**Results:**

There were 14 intracranial complications, 5 in the pre-COVID and 9 in the COVID cohort, including 1 and 5 sigmoid sinus thrombosis, respectively. Out of them, 3 and 5 patients reported a prior history of chronic otitis media, respectively. In COVID period, CVST was more prevalent, with 2 cases (22.2%) presenting solitary CVST, and 3 cases (33.3%) CVST and a simultaneous brain abscess or meningitis. CVST was much more frequent in the COVID period (p < 0.01).

**Conclusions:**

Despite the published data which suggest that CVST is a rare event associated with COVID-19 infection, based on our experience, CVST can be expected as a frequent component of intracranial otogenic complications during COVID-19 pandemic time.

*Trial registration* This research study was conducted retrospectively from data obtained for clinical purposes. We consulted extensively with the Bioethics Committee at Poznan University of Medical Sciences who determined that our study did not need ethical approval. An official waiver of ethical approval was granted from the Bioethics Committee at Poznan University of Medical Sciences.

**Graphical abstract:**

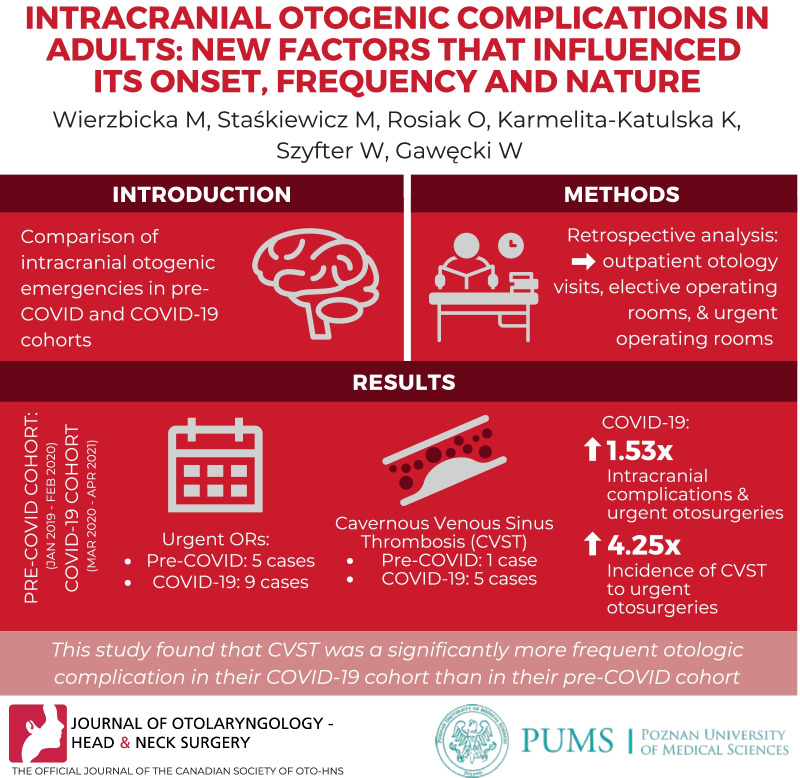

## Background

Coronavirus disease (COVID-19) manifests with a spectrum of symptoms. Nevertheless, otitis has already been highlighted as an unusual presentation that rarely occurs in conjunction with common viral symptoms or as an isolated disorder [1]. The damaging impact of the COVID-19 virus on the inner ear has been explored [2–4]. It was found that cochlea involvement could be frequent even without major general symptoms [3] or sporadic, like radiology-proven acute labyrinthitis [5]. Otitis media and conductive hearing loss was presented as a single case series for its rarity [6].

Acute and chronic otitis should occur at random rates in a given population irrespectively of the COVID-19 pandemic. On the other hand, there are some factors that could potentially influence the incidence of otogenic intracranial complications. Among these are the limited availability of specialist consultations, elevated population morbidity, and the nature of the COVID-19 thromboembolic phenomenon including venous thromboembolism via endothelium rupture [7–9].

Cerebral venous sinus thrombosis (CVST) associated with COVID-19 has been increasingly recognised [10–13] with transverse and sigmoid sinuses as the most common sites, but also CVST spanning multiple sinuses was observed [14]. The frequency of CVST in COVID-19 patients was estimated at 0.0001%-0.08% in recent systematic reviews [15, 16] Although severe cases are usually reported in the elderly and in those with underlying comorbidities, some case reports emphasised that CVST may also occur in young individuals without significant medical history [17]. CVST may be underreported as its presentation can manifest as non-specific clinical syndromes: isolated intracranial hypertension with headache, papilledema, visual deficits or encephalopathy with altered mental status, reduced consciousness, or coma [18]. All aforementioned complaints, although not etiologically bind, resemble symptoms of otogenic sigmoid sinus thrombosis such as headache, dizziness, nausea, and vomiting. Another aspect worth exploring is the extent to which an active or past COVID-19 viral infection can modify the course of middle ear otitis and its complications.

Thus, two questions can be asked amid the COVID-19 pandemic. The first is whether the percentage of complications due to limited access to medical care and neglected ear infections has changed, and the second, whether a possible viral infection could have had an impact on the course of middle ear inflammation and its complications. The research hypothesis assumes that the COVID-19 pandemic has influenced the number and scope of patients admitted as emergency in the tertiary-referral otolaryngology department with intracranial otogenic complications.

The aim of the study is to compare the clinical features of two time cohorts of patients: “pre-COVID-19” and “COVID-19”—admitted as emergency with otogenic complications, with special regard to sigmoid sinus thrombosis.

## Methods

The two cohorts were designed for the purpose of the retrospective analysis. The therapeutic activity of the Department of Otolaryngology at Poznan University of Medical Sciences in two time periods was compared: January 2019–February 2020 and March 2020–April 2021. In the first period, 1434 otological outpatient visits, 509 planned otosurgeries, and 17 urgent otological procedures were performed. In the second period, these were 1150, 566 and, 20 respectively. There were 14 intracranial complications, 5 in the pre-COVID and 9 in the COVID cohort, including 1 and 5 sigmoid sinus thrombosis, respectively.

For the purpose of the study we also analyzed the frequency of intracranial otogenic complications in our hospital in the preceding 5 years. As shown in Table 1. The percentage of CVST in total number of intracranial complications exceeded 40% only once before 2020 and there is no noticeable trend in frequency of complications within those years.

**Table 1:** Incidence of intracranial otogenic complications in time with a special regard to CVST

	All intracranial otogenic complications	CVST	% CVST/All intracranial otogenic complications
2015	6	3	50.00
2016	4	0	0.00
2017	8	0	0.00
2018	4	1	25.00
2019	4	0	0.00
2020	5	2	40.00
2021 (until 04/2021)	5	4	80.00

Planned otosurgeries were defined as tympanoplasty (including cholesteatoma surgeries), stapes surgery, cochlear implants and osseous implants. Urgent otosurgery and inclusion criteria for the otogenic complications group were defined as subacute or chronic otitis media with ear discharge exacerbation, headache, otalgia, and fever at presentation, established diagnosis of intra-temporal (labyrinthitis, facial nerve paresis, mastoiditis) or intracranial complications (meningitis, epidural/subdural/brain abscess, sigmoid sinus/internal jugular vein thrombosis). The time from registration in the emergency room to performing the surgery (antromastoidectomy, wall-up/down technique, abscess drainage, internal jugular vein ligation) ranged from 6 to 24 h; average 14 h, median 10 h. The surgeries were performed by two experienced otosurgeons (MW, WS).

Reverse transcription-polymerase chain reaction (RT-PCR) for SARS-CoV-2 of a nasopharyngeal swab was performed at the emergency department before admission to the ward. IgG antibodies tests for SARS-CoV-2 were performed between the second and fifth day of the hospital stay.

The patients’ data included otological history, character and duration of complaints, comorbidities, neurological status, information on pharmacological prophylaxis, the status of immobility and other risk factors of coagulopathy.

There were no other significant epidemiological factors such as a change in the population size, catchment area, access to health services provided by the hospital or surrounding hospitals, number of patients’ requiring admission to tertiary hospital instead of local one or a change in medical practice.

Relevant laboratory results include white cell count (WBC), platelets (PLT), prothrombin time (PT), activated partial thromboplastin time (aPTT), C-reactive protein (CRP), D-dimer, fibrinogen, and lactic acid dehydrogenase (LDH).

The radiologic diagnosis of CVST was confirmed using the following criteria: (a) Magnetic Resonance Imaging (MRI)/ Magnetic Resonance Venogram (MRV): absence of flow-related signal, (b) T1 weighted sequence with intravenous contrast agent (VIBE, MPRANGE) to confirm the presence of thrombus. In each patient, MRI was performed by 3 T Somatom Skyra Siemens and the structures of the neck were assessed by ultrasonography (Canon APLIO a-series device), with particular attention to the patency of the jugular vein.

The “pre-COVID-19” and “COVID-19” patients with intracranial otogenic complications were compared. The following variables were analysed for both cohorts: age, gender, precise diagnosis of otogenic intracranial complication (meningitis, epidural/subdural/brain abscess, sigmoid sinus/internal jugular vein thrombosis), prior history of ear disease, diagnostic imaging findings and comorbidities classified as cardiovascular, pulmonary, other, and none. The differences in the presented variables for both pre-COVID-19 and COVID-19 cohorts were analysed.

The first outcome measure was the incidence proportion of CVST and intracranial complications of otogenic origin in relation to planned otosurgical procedures or urgent otosurgical procedures in a given time. Secondary outcome measures included the incidence proportions of CVST and intracranial complications in relation to the total number of both emergency and planned outpatient consultations and the total number of planned surgical procedures in both time periods. Both cohorts were analysed for their specificity. The occurrence or absence of CVST among all intracranial complications was also analysed separately.

The incidence proportion was defined as new cases of a disease occurring in a given observation period.

$${\text{incidence}}\,{\text{proportion = }} \frac{{{\text{new}}\,{\text{cases}}\,{\text{of}}\,{\text{the}}\,{\text{disease}}}{}}{{\text{number}}\,{\text{of}}\,{\text{persons}}\,{\text{in}}\,{\text{the}}\,{\text{population}}\,{\text{in}}\,{\text{the}}\,{\text{observed}}\,{\text{period}}} \times 100\%$$

For the statistical analysis, the STATISTICA 13.1 software (Dell, U.S.A) was utilised. Nominal variables were compared between the groups in contingency tables using Fisher’s exact test; in cases where a particular cell count was null, the Haldane-Anscombe correction was applied. For 2 × 3 and 2 × 4 tables, the Freeman-Halton extension to the Fisher’s exact test was utilised. Continuous variables distribution was checked for normality using the Shapiro–Wilk test. All continuous variables were non-normally distributed, therefore the Mann–Whitney U test for non-parametric data was used to compare the groups. Where possible, the Odds Ratio with a 95% confidence interval was calculated. The alpha for all statistical tests was set at 0.05.

## Results

The intracranial complications and urgent otosurgeries incidence proportion calculated for the pre-COVID period of 2019/2020 was 29.41% and 45% for the COVID period, which is a 1.53 × increase. The incidence proportion of CVST to urgent otosurgeries in the pre-COVID period was 5.88%, while in the COVID period it was calculated at 25%, a 4.25 × increase. The data regarding calculated incidence proportions in regard to different denominators is summarised in Table 2.

**Table 2:** Number of urgent and planned procedures regarding all ENT and otologic surgeries in two time cohorts

Parameter	Time cohort		COVID-19/Pre-COVID-19
	Pre-COVID-19	COVID-19	
All otogenic intracranial complications (2)	5	9	180%
Sigmoid sinus thrombosis (1)	1	5	500%
All ENT Surgical procedures (P1)	1520	1371	90%
All ENT-related Emergency consultations (P2)	1260	1232	98%
Planned otosurgical procedures (P3)	509	566	111%
Urgent otosurgical procedures (P4)	17	20	118%
Out-patient otological consultations (P5)	1434	1150	80%
Incidence Proportion 1 (1/P1)	0.07%	0.36%	5.14
Incidence proportion 2 (2/P1)	0.33%	0.66%	2.0
Incidence proportion 3 (1/P2)	0.08%	0.41%	5.13
Incidence proportion 4 (2/P2)	0.4%	0.73%	1.83
Incidence proportion 5 (1/P3)	0.2%	0.88%	4.4
Incidence proportion 6 (2/P3)	0.98%	1.59%	1.62
Incidence proportion 7 (1/P4)	5.88%	25%	4.25
Incidence proportion 8 (2/P4)	29.41%	45%	1.53
Incidence proportion 9 (1/P5)	0.07%	0.43%	6.14
Incidence proportion 10 (2/P5)	0.35%	0.78%	2.23

Incidence proportions were calculated for the incidence of sigmoid sinus thrombosis (1) and otogenic intracranial complications (2)

**Fig. 1:**
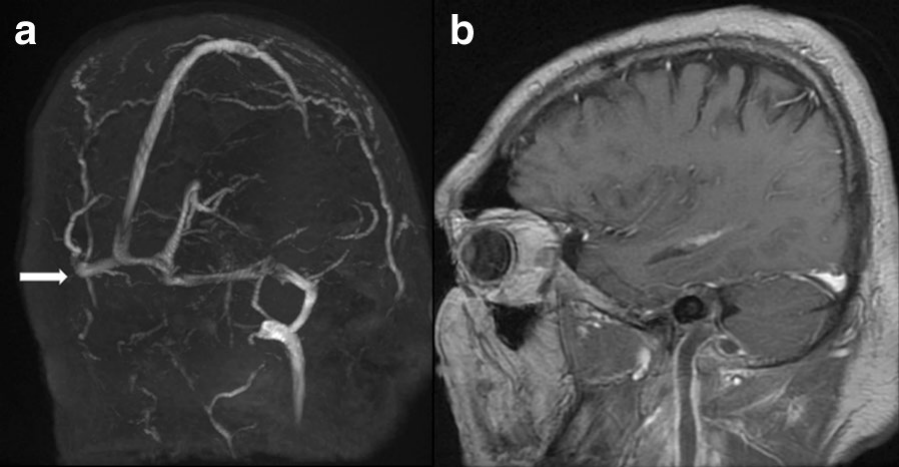
MRV image (**a**), no flow in the right transverse and sigmoid sinus also no flow in jugular vein; T1 VIBE sagittal (**b**) image with contrast, corresponding thrombus in jugular vein and sigmoid sinus

Presence of CVST in the sigmoid sinus in pre-COVID and COVID-19 cohorts was seen in 1 and 5 cases (Tables 2, 3), respectively. Involvement of the deep venous system was observed in 2 cases, having internal jugular and subclavian vein thrombosis (Fig. 1a, b). No intracranial bleeding was seen on initial neuroimaging in the patients.

**Table 3:** Comparison of nominal and non-nominal characteristics of patients divided into two time cohorts

	Time cohort		OR (95% CI)	Probability
	Pre-COVID-19	COVID-19		
*Sex*				
Male	5 (100%)	8 (88.9%)	0.56 (0.02; 15.06)	p = 1*
Female	0	1 (11.1%)		
*Venous sinus thrombosis*				
Absent	4 (80%)	4(44.4%)	5 (0.38; 64.38)	p = 0.3
Present	1 (20%)	5 (55.6%)		
*Comorbidities*				
Other	1 (20%)	0	N/A	p = 0.002**
Cardiovascular	1 (20%)	5 (55.6%)		
Pulmonary	1 (20%)	2 (22.2%)		
None	2 (40%)	2 (22.2%)		
*Intracranial complications*				
Meningitis	2 (40%)	1 (11.1%)	N/A	p = 0. 0.009**
Brain abscess	2 (40%)	3 (33.3%)		
Venous sinus thrombosis	0	2 (22.2%)		
Brain abscess or meningitis with sinus thrombosis	1 (20%)	3 (33.3%)		
*Prior history of chronic otitis*				
Yes	3 (60%)	5 (55.56%)	1.2 (0.13; 11.05)	p = 1^F^
No	2 (40%)	4 (44.4%)		
*Risk factors for coagulopathies*				
Yes	1 (25%)	7 (12.5%)	0.05 (0.002; 1.04)	p = 0.067^F^
No	3 (75%)	1 (87.5%)		

	Median (IQR)	Median (IQR)		
Age	32 (19)	50 (22)	N/A	p = 0.547^UM^
PT	14.75 (1.35)	15.1 (3)	N/A	p = 0.699^UM^
INR	1.35 (0.13)	1.39 (0.29)	N/A	p = 0.643^UM^
APTT	31 (2.5)	32 (7)	N/A	p = 0.816^UM^
WBC	16.63 (4.92)	16.45 (6.07)	N/A	p = 0.699^UM^
PLT	235 (224)	377 (399)	N/A	p = 0.316^UM^
Fibrinogen	273 (378.5)	336 (299)	N/A	p = 0.316^UM^
D-dimers	0.12 (0.56)	0.77 (1.3)	N/A	p = 0.247^UM^
CRP	111.05 (164.8)	147.4(180.8)	N/A	p = 0.758^UM^
LDH	134.5 (127)	177 (44)	N/A	p = 0.316^UM^
Total number of patients	N = 5	N = 9		

Underline values indicate significant (*p* < 0.05)

N/A – not applicable; IQR – Interquartile ratio; OR – Odds Ratio; 95% CI – Confidence Interval; PT – prothrombin time; INR – International normalised ratio; APTT – activated partial thromboplastin time; WBC – White Blood Cell Count; PLT – Platelets; CRP – C-reactive protein levels; LDH – Lactic Acid Dehydrogenase

*Haldane-Anscombe correction to Fisher’s exact test was applied

**Fisher’s exact test after Haldane-Anscombe correction with Freeman-Halton extension for 2 × 4 contingency tables was used

^F^Fisher’s exact test was used

^UM^Mann–Whitney U test for non-parametric data was utilised

In the COVID-19 cohort, all 5 patients with sigmoid sinus thrombosis polymerase chain reaction testing of a nasopharyngeal swab was negative for COVID-19 at admission. None of the patients displayed symptoms related to COVID-19 infection. One patient tested positive in a PCR test while presenting no typical symptoms of COVID-19 on the 10th day of stay, before discharge. Nevertheless an antibody testing revealed high IgG titers in serum in 3 others of the 5.

Symptoms related to middle ear infection complicated by sigmoid sinus thrombosis included fever (1/5), headache (5/5), earache (1/5), purulent discharge (2/5), disturbances of consciousness (2/5), facial nerve paresis (1/5), epileptic seizure (1/5). In otoscopy, tympanic membranes were erythematous and bulging (4/5), erythema or tenderness overlying the mastoid processes, protrusion of the pinna (1/5) was noted. One had a tympanic membrane perforation. Most patients had hearing loss; conductive hearing loss and mild sensorineural hearing loss.

In total, there were 5 intracranial complications in the pre-COVID cohort, which included 2 cases (40%) of meningitis, 2 cases (40%) of brain abscesses, and 1 case (20%) presenting a brain abscess and CVST simultaneously (Table 3). In the COVID period, CVST was more prevalent, with 2 cases (22.2%) presenting solitary CVST of otogenic origin, and 3 cases (33.3%) CVST and a simultaneous brain abscess or meningitis, resulting in a total of 5 cases with CVST (55.5%). CVST was more frequent in the COVID cohort (p < 0.01). In the analysis of coexistent chronic diseases, 55.6% of patients in the COVID-19 cohort had cardiovascular comorbidities, while in the pre-COVID-19 cohort, only 1 patient had a cardiovascular disorder (20%) (p < 0.01) (Table 4). Three patients of that cohort reported a prior history of chronic otitis (60%), whereas in the COVID-19 cohort, 5 patients had a prior history of otologic disease (55.56%).

**Table 4:** Comparison of nominal and non-nominal characteristics between patients with confirmed venous sinus thrombosis and the absence of thrombus

	Venous sinus thrombosis		OR (95% CI)	Probability
	Absent	Present		
*Sex*				
Male	7 (87.5%)	6 (100%)	2.6 (0.09; 75.5)	p = 1*
Female	1 (12.5%)	0		
*Comorbidities*				
Other	1 (12.5%)	2 (33.3%)	N/A	P = 0.005**
Cardiovascular	4 (50%)	2 (33.3%)		
Pulmonary	0	1 (16.67%)		
None	3 (37.5%)	1 (16.67%)		
*Prior history of chronic otitis*				
Yes	5 (62.5%)	3 (50%)	5 (0.07; 5.136)	p = 1^F^
No	3 (37.5%)	3 (50%)		
*Risk factors for coagulopathies*				
Yes	4 (50%)	4 (66.6%)	0.5 ( 0.06; 4.47)	p = 0.627^F^
No	4 (50%)	2 (33.3%)		

	Median (IQR)	Median (IQR)		
Age	41.5 (21)	48.5 (23)	N/A	p = 0.846^UM^
PT	14.15 (1.9)	17 (2)		p = 0.048^UM^
INR	1.29 (0.17)	1.57 (0.19)		p = 0.040^UM^
APTT	30.5 (3)	36 (9)		p = 0.162^UM^
WBC	16.63 (7.53)	16.45 (3.05)		p = 0.608^UM^
PLT	285.5 (345)	377 (438)		p = 0.826^UM^
Fibrinogen	285 (59)	600 (4)		p = 0.034^UM^
D-dimers	0.12 (0.16)	1.5 (1.18)		p = 0.010^UM^
CRP	72.7 (198.85)	165.8 (69.3)		p = 0.305^UM^
LDH	145.5 (84)	48.5 (34)		p = 0.213^UM^
Total number of patients	N = 8	N = 6		

Underline values indicate significant (*p* < 0.05)

N/A – not applicable; IQR – Interquartile ratio; OR – Odds Ratio; 95% CI – Confidence Interval; PT – prothrombin time; INR – International normalised ratio; APTT – activated partial thromboplastin time; WBC – White Blood Cell Count; PLT – Platelets; CRP – C-reactive protein levels; LDH – Lactic Acid Dehydrogenase

*Haldane-Anscombe correction to Fisher’s exact test was applied

**Fisher’s exact test after Haldane-Anscombe correction with Freeman-Halton extension for 2 × 4 contingency tables was used

^F^Fisher’s exact test was used

^UM^Mann–Whitney U test for non-parametric data was utilised

In terms of laboratory findings, all patients exhibited elevated CRP and WBC, however upon comparing the cohorts no statistically significant differences were noted in any of the analysed laboratory parameters or median age.

A separate analysis was performed for cases presenting with CVST in an attempt to identify risk factors for developing venous thrombosis, which was more prevalent in the COVID-19 cohort. Neither known risk factors for coagulopathies (obesity, alcoholism, smoking, or immobility classified as “yes” or “no”), sex, nor prior history of chronic otitis showed statistically significant differences between the groups, and thus could not be considered as risk factors for CVST in the study population.

Considering laboratory parameters, patients with CVST showed increased PT and INR levels as well as elevated D-Dimer and Fibrinogen concentrations (p < 0.05) (Table 4).

**Fig. 2:**
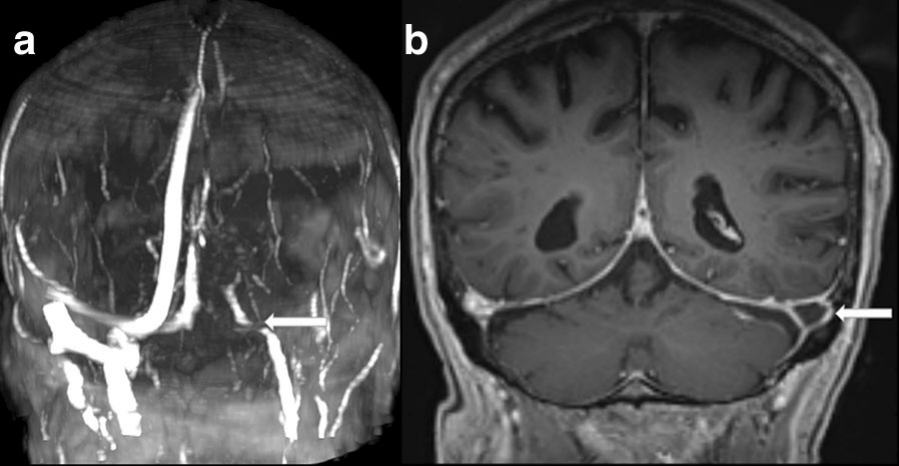
**a** MRV—no flow in sigmoid and transverse sinus, **b** T1 MPRANGE image coronal reconstruction with contrast, thrombus in left sigmoid sinus

The absence of flow-related signal was seen in 6 cases of CVST (100%) (Figs. 1a, 2a) while all cases without CVST presented flow in the MRV.

## Discussion

We present, the first to our knowledge, this cohort analysis of the therapeutic activity of the emergency ENT Tertiary referral centre during the COVID-19 pandemic in terms of urgent otosurgery. We base our observations and conclusions on a comparison of the patients admitted in the time periods of January–February 2019/2020 and March–April 2020/2021. In reviewing the medical activities of the ENT department, we have summarised the numbers of services provided. The numbers of otological outpatient visits and planned otological surgeries were comparable and oscillated slightly higher; also, an increased intracranial complication count (5 versus 9) and decreased overall operation capacity (1520 versus 1371) is stated (Table 2).

In addition to quantitative analysis, the qualitative change in patients’ medical history and course of the disease is of key importance. Intracranial complications as a result of otogenic infections occur despite the antibiotic era [19, 20], but are extremely rare with no background history of otological problems. Cholesteatoma has been reported to be the most common cause of otogenic sigmoid sinus thrombosis, but is a rare complication of acute otitis media [20]. We found a completely different clinical course in 3/5 patients in the COVID-19 cohort. Acute otitis media transforming into a subacute process, resulting in insidious CVST complications within 3–4 weeks from ear pain onset was presented. The treatment in patients with acute otitis media did not differ in pre-COVID and COVID time. All the patients had antibiotic treatment, nevertheless in COVID-19 era it was partially prescribed via telemedicine. Our findings have not been reflected in the current literature.

The pathognomonic features of sigmoid sinus and mastoid vein thrombosis are retroauricular oedema and pressing pain (Griesinger sign) [19, 20]. Therefore, the Griesinger sign can be considered as a possible warning of a forthcoming complication. In our patients’ series, the predominant signs were not typical, with headache as an isolated symptom. Thus, in 5/9 patients, the first contact physician was not an ENT specialist, but a neurologist.

During the pandemic, part of the population developed SARS-Cov-2 infection asymptomatically but later presented varied post-COVID syndrome or with unproven immunodeficiencies. According to the literature, a significant portion of CVST patients displayed only mild to moderate severity of COVID-19 infection, thus indicating that a hypercoagulable state may be present even in mild infection [21]. In COVID-19 cohort no patient had severe infection, 1 mild, 1 asymptomatic with positive PCR test during hospital stay and 6 had only elevated IgG. None of the CVST patients had clinical evidence of COVID-19 infection. We believe that a portion of patients had low grade COVID infections in the past and were more likely to display a hypercoagulable state while another portion of patients had delays in seeking medical care thus leading to these complications. The length of hypercoagulable state as well as its dependance on the course of COVID-19 infection needs further study.

Additionally our cohort analysis of the otogenic intracranial complications clearly showed that the COVID-19 cohort had more cardiovascular comorbidities than the pre-COVID, which is reflected in literature [16].

Our study has several limitations, which include the retrospective nature of the pre-COVID cohort and limited number of cases, which does not allow us to analyse the risk factors for CVST in COVID-19 time. Our data does not include detailed information about follow-up because patients from a distance of more than 100 km report for a control at the place of residence and in the period that followed, none came with further ailments.

The strength of our study is that it provides—the first to our knowledge—comparative data from an Otosurgery Department and unique middle ear complication cohorts with special regard to CVST in COVID-19 compared to pre-COVID patients. However, the major advantages of our study include new data on the divergent course of acute otitis media with latent nature and insidious intracranial complication onset.

## Conclusions

To summarise, the published data of patients with CVST and COVID-19, suggest that acute CVST is a rare event associated with COVID-19 infection. But based on our experience, CVST can be expected as a frequent component of intracranial otologic complications, both with chronic and acute middle ear origin.

## Data Availability

The datasets generated and analysed during the current study are not publicly available but are available from the corresponding author on reasonable request.

## References

[1] Fidan V (2020). New type of corona virus induced acute otitis media in adult. Am J Otolaryngol.

[2] Viola P, Ralli M, Pisani D (2020). Tinnitus and equilibrium disorders in COVID-19 patients: preliminary results. Eur Arch Otorhinolaryngol.

[3] Mustafa MWM (2020). Audiological profile of asymptomatic Covid-19 PCR-positive cases. Am J Otolaryngol.

[4] Elibol E (2020). Otolaryngological symptoms in COVID-19. Eur Arch Otorhinolaryngol.

[5] Perret M, Bernard A, Rahmani A, Manckoundia P, Putot A (2021). Acute labyrinthitis revealing COVID-19. Diagnostics.

[6] Raad N, Ghorbani J, Mikaniki N, Haseli S, Karimi-Galougahi M (2021). Otitis media in coronavirus disease 2019: a case series. J Laryngol Otol.

[7] Guendouz C, Quenardelle V, Riou-Comte N (2021). Pathogeny of cerebral venous thrombosis in SARS-Cov-2 infection: case reports. Medicine (Baltimore).

[8] Dakay K, Cooper J, Bloomfield J (2021). Cerebral venous sinus thrombosis in COVID-19 infection: a case series and review of the literature. J Stroke Cerebrovasc Dis Off J Natl Stroke Assoc.

[9] Speeckaert MM, Speeckaert R, Delanghe JR (2020). Potential underlying mechanisms of cerebral venous thrombosis associated with COVID-19. J Neuroradiol.

[10] Khazaei M, Karimi K, Sedighi P, Khazaei S (2021). Cerebral sinus thrombosis secondary to SARS-CoV-2 infection. Case Rep Neurol Med.

[11] Tveit L, Enriquez B, Tennøe B (2020). Cerebral venetrombose etter covid-19. Tidsskr Den Nor Legeforening.

[12] Cavalcanti DD, Raz E, Shapiro M (2020). Cerebral venous thrombosis associated with COVID-19. AJNR.

[13] Medicherla CB, Pauley RA, de Havenon A, Yaghi S, Ishida K, Torres JL (2020). Cerebral venous sinus thrombosis in the COVID-19 pandemic. J Neuro-Ophthalmol Off J North Am Neuro Ophthalmol Soc.

[14] Abdalkader M, Shaikh SP, Siegler JE (2021). Cerebral venous sinus thrombosis in COVID-19 patients: a multicenter study and review of literature. J Stroke Cerebrovasc Dis.

[15] Baldini T, Asioli GM, Romoli M (2021). Cerebral venous thrombosis and severe acute respiratory syndrome coronavirus-2 infection: a systematic review and meta-analysis. Eur J Neurol.

[16] Hinduja A, Nalleballe K, Onteddu S, Kovvuru S, Hussein O (2021). Impact of cerebral venous sinus thrombosis associated with COVID-19. J Neurol Sci.

[17] Klein DE, Libman R, Kirsch C, Arora R (2020). Cerebral venous thrombosis: atypical presentation of COVID-19 in the young. J Stroke Cerebrovasc Dis.

[18] Rigamonti A, Mantero V, Piamarta F, Spena G, Salmaggi A (2021). Cerebral venous thrombosis associated with coronavirus infection: an underestimated entity?. Neurol Sci.

[19] Raja K, Parida PK, Alexander A, Surianarayanan G (2018). Otogenic lateral sinus thrombosis: a review of fifteen patients and changing trends in the management. Int Arch Otorhinolaryngol.

[20] Ropposch T, Nemetz U, Braun EM, Lackner A, Tomazic PV, Walch C (2011). Management of otogenic sigmoid sinus thrombosis. Otol Neurotol Off Publ Am Otol Soc Am Neurotol Soc Eur Acad Otol Neurotol.

[21] Tu TM, Goh C, Tan YK (2020). Cerebral venous thrombosis in patients with COVID-19 infection: a case series and systematic review. J Stroke Cerebrovasc Dis.

